# Author Correction: Dynamic causal modelling of immune heterogeneity

**DOI:** 10.1038/s41598-021-95594-3

**Published:** 2021-08-03

**Authors:** Thomas Parr, Anjali Bhat, Peter Zeidman, Aimee Goel, Alexander J. Billig, Rosalyn Moran, Karl J. Friston

**Affiliations:** 1 Wellcome Centre for Human Neuroimaging, Queen Square Institute of Neurology, London, UK; 2 Royal Stoke University Hospital, Stoke-on-Trent, UK; 3 UCL Ear Institute, University College London, London, UK; 4 Centre for Neuroimaging Science, Department of Neuroimaging, IoPPN, King’s College London, London, UK

Correction to: *Scientific Reports* 10.1038/s41598-021-91011-x, published online 31 May 2021.

The original version of this Article contained an error in the Acknowledgments section.

“AB is supported by a Medical Research Council doctoral studentship [D79/543369/D-OTH/170890].”

now reads:

“AB is supported by a Medical Research Council doctoral studentship [MR/N013867/1].”

The original Article has been corrected.

